# Molecular and morphological alterations in breast tissue of transgender patients undergoing dihydrotestosterone therapy

**DOI:** 10.1371/journal.pone.0325034

**Published:** 2025-07-29

**Authors:** Jinho Lee, Maryam Foroughi, Vanderlene Kung, Rabeka Ali, Saachi Parikh, Ann McMonigal, Yun Yu, Austin Nguyen, Gabriel Zangirolani, Lina Gao, Joanna Pucilowska, Ozlen Saglam

**Affiliations:** 1 Department of Biology, Knight Cancer Center, Portland, Oregon, United States of America; 2 Department of Pathology and Laboratory Medicine, Oregon Health and Science University, Portland, Oregon, United States of America; 3 Department of Biostatistics, Knight Cancer Center, Portland- Oregon, United States of America; 4 Department of Immune Monitoring and Therapeutic Analytics, Knight Cancer Center, Portland, Oregon, United States of America; Mie University Hospital: Mie Daigaku Igakubu Fuzoku Byoin, JAPAN

## Abstract

Many patients undergoing gender-affirming surgery (GAS) opt for reconstructive procedures rather than total mastectomy to achieve a more masculine chest contour. The impact of dihydrotestosterone (DHT) treatment on breast tissue remains unclear. This study evaluates the morphological changes and protein expression levels in breast tissue associated with hormonal and molecular pathways in patients receiving short-term or long-term DHT treatment before GAS. A total of 230 breast tissue samples were categorized into three groups: nontreatment, short-term treatment (STT, < 12 months), and long-term treatment (LTT, ≥ 12 months). Paired samples (n = 33) were stained for estrogen receptor (ER) and androgen receptor (AR). NanoString Digital Spatial Profiling (DSP) analysis was conducted on a subset (n = 17), including two incidental breast cancer (BC) cases. Among morphological parameters assessed, atrophy and secretory changes differed significantly among groups. In the LTT group, ER-alpha expression was elevated in lactiferous ducts, while AR H-scores were higher in both STT and LTT groups. ER and AR expression levels were strongly correlated in the STT and LTT groups (r = 0.93–0.99). DSP analysis revealed increased ER expression in the treated groups and higher AR expression in peripheral lobules of the LTT group (log2FC = 1.3, p = 0.03). Ki-67, CDK6, and CD45 levels decreased in the LTT group, while INPP4B and BCL6 increased. DHT treatment leads to significant morphological and molecular changes in both benign and cancerous breast tissue. Altered expression of biomarkers such as INPP4B and CD45 in the LTT group and breast cancer samples suggests a potential role in BC development, warranting further investigation.

## Introduction

Gender dysphoria refers to the psychological distress or discomfort arising from a mismatch between an individual’s gender identity and the sex assigned at birth. Population-based surveys estimate that 580 out of every 100,000 people in the United States identify as transgender [1]. A meta-analysis of individuals seeking clinical services reported prevalence rates of 6.8 per 100,000 for transwomen (Male-to-Female, MTF) and 2.6 per 100,000 for transmen (Female-to-Male, FTM) [2]. In recent years, there has been a notable increase in gender-affirming surgeries (GAS), with breast and chest surgeries accounting for the majority (57%), followed by genital reconstruction (35%) and various facial and cosmetic procedures [3].

In FTM patients, several factors influence the decision-making process regarding the type of chest reconstruction, including age, breast size, breast ptosis, skin quality, body mass index (BMI), and most importantly, the patient’s relative risk for breast cancer (BC) [4]. The goal of chest reconstruction is to reduce breast volume and skin excess [5]. Depending on the technique, some central (large ductal epithelium) and peripheral breast tissue may remain after the procedure. Dihydrotestosterone (DHT) therapy plays a crucial role in gender-affirming care, but its impact on breast tissue remains unclear.

FTM patients typically receive testosterone therapy at high doses to achieve endogenous testosterone levels comparable to those of cisgender males [6]. Studies from large centers have documented common morphological changes after testosterone treatment, including lobular atrophy, ectatic ducts, and changes resembling the fibrous stage of gynecomastia [7].

This study aims to examine the short- and long-term effects of DHT therapy on breast morphology, hormonal expression, and key biological pathway markers in transgender men. First, an H&E slides review (n = 230) was performed to evaluate benign and precancerous breast lesions systematically. Samples were categorized into three groups: nontreatment (patients not exposed to DHT before surgery), short-term treatment (<12 months before surgery), and long-term treatment (≥12 months before surgery). Of the 230 mastectomy specimens, 33 paired samples were stained for estrogen receptor (ER) and androgen receptor (AR) to assess expression differences in the lactiferous ductal epithelium and peripheral lobules across the three groups. Finally, NanoString Digital Spatial Profiling analysis was performed on a subset (n = 17), including two incidental BC samples, to investigate markers associated with pan-tumor pathways, cell cycle regulation, oncogenic signaling, and immune-cell profiling.

## Materials and methods

After receiving Oregon Health and Science University Review Board (IRB) approval, archival samples collected between January 1, 2006, and December 31, 2023, were retrieved and analyzed on September 1, 2023. The collected data were fully anonymized to remove patient identifiers and included demographic information such as age, type of surgeries performed, duration of DHT treatment, and BMI. For benign cases (n = 230), an average of seven H&E slides per sample were reviewed (range: 4–13). All samples were evaluated for the presence or absence of focal or diffuse lobular atrophy and secretory changes, including apocrine snouts, luminal cell separation, shedding into the ductal lumen, intracytoplasmic vacuoles, luminal secretions, and fibrocystic changes. Myoepithelial cell hyperplasia in the large ductal epithelium, defined as the presence of more than one layer of myoepithelial cells, was also assessed. Additionally, incidental precursor breast lesions, such as atypical ductal or lobular hyperplasia, were noted. Findings from patients under 18 were compared with those from adults. BMI was categorized into five groups (<18.5, 19–24.9, 25–29.9, 30–34.9, and >35) and analyzed for associations with morphological features and hormone expression levels.

### Immunohistochemistry

Immunostaining for ER-alpha and AR was performed on paired sections for each sample: one from central breast tissue (lactiferous ducts) and one from peripheral tissue (lobules), using the Ventana Discovery XT Automated System (Ventana Medical Systems, Tucson, AZ). Slides were deparaffinized using a preparatory solution on the automated system. Rabbit monoclonal antibodies were used for AR (SP107, Cell Marque) and ER (SP1, Roche). Heat-induced antigen retrieval was performed at pH 6, with AR for 60 minutes and ER for 30 minutes. Counterstaining with hematoxylin and bluing was applied for 4 minutes, followed by coverslipping.

The H-score (0–300) was used for semi-quantitative assessment of ER and AR expression levels. The score was calculated by multiplying the percentage of positively stained cells (0–100%) by nuclear staining intensity (0–3+). Paired samples from central and peripheral breast tissue were compared across the three groups. Immunostaining scores were correlated with staining intensities obtained via computer-assisted image analysis.

### Computer-assisted image analysis (CAIA)

Computer-assisted semi-quantitative analysis of staining intensity was performed on all ER and AR immunohistochemically stained sections using ImageJ Fiji software following the protocol described by Crowe et al [8]. Briefly, ten, 100X magnification fields of view were selected per immunohistochemistry-stained section and deconvoluted with Fiji using the “H DAB (hematoxylin and 3,3’-diaminobenzidine)” vector. Thresholds were selected to include all epithelial cell nuclei, and individual nuclei were segmented using the “binary watershed” function. Intensity values were divided by the number of nuclei measured to obtain normalized staining intensities.

### Statistical analysis of immunostaining results

Statistical analyses were conducted using IBM SPSS Statistics, version 29.0.1.0 (IBM Corp., Armonk, NY, USA). Differences in H-scores among the three groups and within paired samples were evaluated using analysis of variance (ANOVA). A p-value of <0.05 was considered statistically significant. Tukey’s post hoc test was performed to identify significantly different H-scores between specific treatment groups. Associations between treatment groups and morphological features, including atrophy, secretory changes, myoepithelial hyperplasia, and fibrocystic changes, were examined using chi-square and Fisher’s exact tests. Age at surgery and BMI were also analyzed for association with morphological features. Pearson’s product-moment correlation coefficient was used to assess correlations between H-scores and the correlation between pathologists and CAIA.

### NanoString’s GeoMx digital spatial profiler (DSP)

Formalin-fixed, paraffin-embedded samples were prepared with DSP protein panels targeting 83 proteins. The custom panel included molecules related to cell cycle and DNA damage signaling. DNA oligo barcodes were cleaved by UV light and collected from three regions of interest (ROIs) per sample. Seventeen samples—five from each of the nontreatment, short-term treatment (STT), and long-term treatment (LTT) groups—along with two incidental invasive ductal carcinoma samples, were included in the DSP analysis. In the cancer samples, both neoplastic tissue and surrounding benign epithelium were collected. Each ROI was segmented into two areas of interest (AOIs) based on the epithelial marker (PanCK+) and its 20 µm periphery for the stromal region. DNA oligo samples were hybridized with fluorescent color-barcoded hybridization codesets and read in the nCounter MAX system for molecule counts. Data were re-imported into the DSP system and normalized using all target geomean analysis method. Further analyses were conducted using Rv4.2.2 and figures were generated using R packages ggplot (version 3.4.2) and Complex Heatmap v2.14.0. Heatmap distances were calculated using Euclidean distance, and clustering was performed using complete-linkage clustering. Comparisons between conditions were performed using a pairwise Wilcoxon rank sum test with Benjamini-Hochberg correction.

## Results

### Clinical and morphological results

The mean patient age was 25 years (range: 15–58), with 24 patients (10%) under the age of 18. Of the surgeries performed, 180 patients (78%) underwent nipple-sparing procedures, and 50 (22%) had simple mastectomies. Most patients received DHT treatment before surgery, ranging from 4 to 60 months. In contrast, 35 patients (15%) did not receive hormonal therapy before surgery. Twenty-eight patients (12%) underwent short-term treatment, and 167 (73%) underwent long-term treatment. Regarding BMI, 47 patients (20%) had a BMI over 35, and 3 patients (1.3%) had a BMI below 18.5. Neither BMI nor age at surgery were associated with specific morphological features.

**Fig 1:**
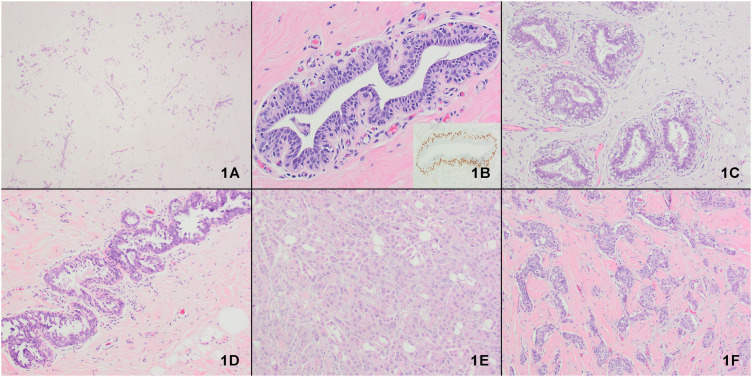
Morphologic changes after dihydrotestosterone (DHT) treatment.1A: Diffuse lobular atrophy (20x). 1B: Myoepithelial cell hyperplasia (200x). The inset demonstrates p63 immunostaining in the myoepithelial cells (400x). 1C: Cytoplasmic clearing in myoepithelial cells (40x). 1D: Luminal cell separation and cell shedding (40X). IE: Incidental invasive carcinoma with apocrine features (200x). 1F: Incidental invasive ductal carcinoma, NOS (200x).

The most common benign findings included fibrocystic changes (13%) and usual ductal hyperplasia (13%), followed by fibroadenomatoid change (4%), microcalcifications (3%), and focal pregnancy-like changes (2.6%). Atypical lobular hyperplasia, flat epithelial atypia, and apocrine atypia were each observed in one sample. Of the 230 samples, 175 (76%) exhibited focal or diffuse lobular atrophy (Fig 1). Atrophy was observed in 20% of samples from patients without DHT treatment, compared to 89% in the STT group and 86% in the LTT group (p < 0.00001). Secretory changes were most prevalent in the LTT group (77%, 128/167), followed by the STT group (64%, 18/28), and least in the nontreatment group (34%, 12/35). Prominent features in the LTT group included apocrine snouting, luminal cell separation, and shedding (Fig 1). The secretory changes were significantly associated with DHT treatment (p < 0.00001). In addition to myoepithelial hyperplasia, a few cases showed myoepithelial cell clearing. The prevalence of other morphological features, such as fibrocystic changes and myoepithelial hyperplasia, did not differ among the groups.

The ages of the patients with incidental carcinoma were 44 and 33 years. The first patient was a carrier of Hepatitis C and Human Immunodeficiency Virus. There was a family history of breast cancer in his paternal grandmother, who was diagnosed in her 80s. He had not undergone a mammogram in the past and had used testosterone for 15 years before the surgery. His ductal carcinoma had micropapillary and apocrine features (Fig 1) and measured 1.8 cm at its greatest dimension. The cancer was strongly positive for ER, progesterone receptor (PR), and AR. HER2/neu was equivocal by immunohistochemistry and was not amplified by FISH. The second patient did not have a family history of breast cancer. He had received testosterone therapy for 1.5 years before the surgery. The tumor was diagnosed as grade 2 invasive ductal carcinoma, not otherwise specified, measuring 4 mm. It was strongly positive for ER, PR, and AR, with negative HER2/neu testing.

### Immunohistochemistry

Paired central and peripheral sections from 33 cases were analyzed, comprising 9 samples from the nontreatment group, 7 from the STT group, and 17 from the LTT group. The mean ER expression in the large ductal epithelium (central breast tissue) was 8 (Standard deviation (SD): 5.6), 95 (SD: 114), and 118 (SD: 99) for the nontreatment, STT, and LTT groups, respectively. The mean ER-alpha expression differed across the groups, with significantly higher ER expression in the LTT group compared to the nontreatment group (p = 0.01). Peripheral lobular ER expression was recorded at higher levels than central breast tissue, with H-scores of 53 (SD: 44.2), 158 (SD: 81.8), and 142 (SD: 108) across the groups. However, no significant differences in peripheral ER expression were observed among the treatment groups.

The mean AR expression in the lactiferous ducts was 49 (SD: 56.7), 99 (SD: 106), and 142 (SD: 96.7) for the nontreatment, STT, and LTT groups, respectively. Similar to ER expression, the mean AR expression in the lactiferous ducts was significantly higher in the LTT group compared to the nontreatment group (p = 0.04). In contrast to ER expression results, AR expression differed in peripheral breast tissue. Both STT and LTT groups had significantly higher H-scores than the nontreatment group (p = 0.04 and 0.01, respectively). The mean peripheral H-scores for AR expression increased from 34 (SD: 30.4) in the nontreatment group to 150 (SD: 99.1) in the STT group and 156 (SD: 103) in the LTT group. No difference was observed between the STT and LTT groups. The mean H-scores for ER expression, assessed by CAIA, were 9,060 in the central breast tissue and 11,028 in the peripheral tissue. Similarly, the mean H-scores for AR expression were 11,206 and 10,939, respectively. H-scores recorded by pathologists and CAIA showed a strong correlation (r = 0.8 and 0.9).

**Fig 2:**
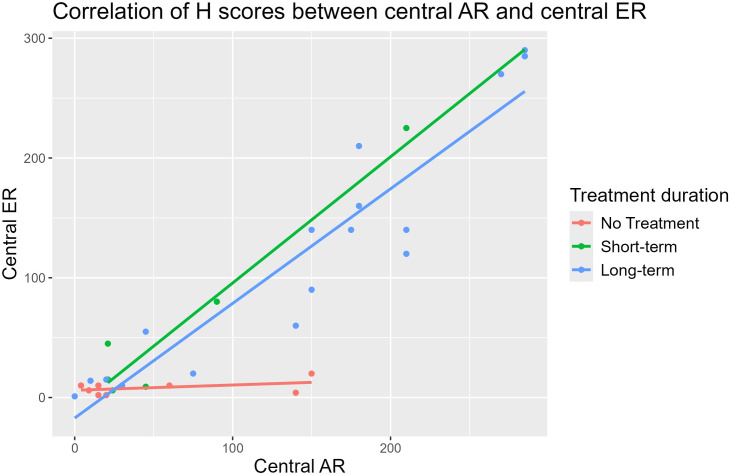
Correlations for H-scores of AR and ER expression in the lactiferous ducts {STT (r = 0.99) and the LTT groups (r = 0.93)}.

**Fig 3:**
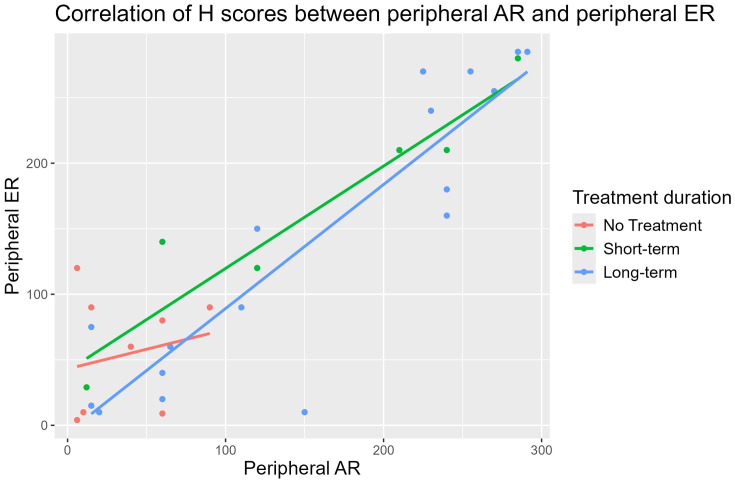
Correlation of H-scores of AR and ER expression in the peripheral lobules {STT (r = 0.95) and the LTT groups (r = 0.90)}.

In the central breast tissue, ER and AR expressions were strongly correlated in the STT (r = 0.99) and the LTT groups (r = 0.93), but a moderate correlation was observed in the nontreatment group (r = 0.44). In the peripheral lobules, ER and AR expressions were also strongly correlated in the STT group (r = 0.95) and the LTT group (r = 0.90), with a weak correlation in the nontreatment group (r = 0.21). (Fig 2 and 3). When comparing ER expression between the large ductal epithelium and peripheral lobules, the correlations were strong in the STT (r = 0.74) and LTT (r = 0.71) groups. AR expression correlations were moderate in the STT (r = 0.60) and LTT (r = 0.58) groups.

### NanoString DSP analysis

#### Benign samples.

**Fig 4:**
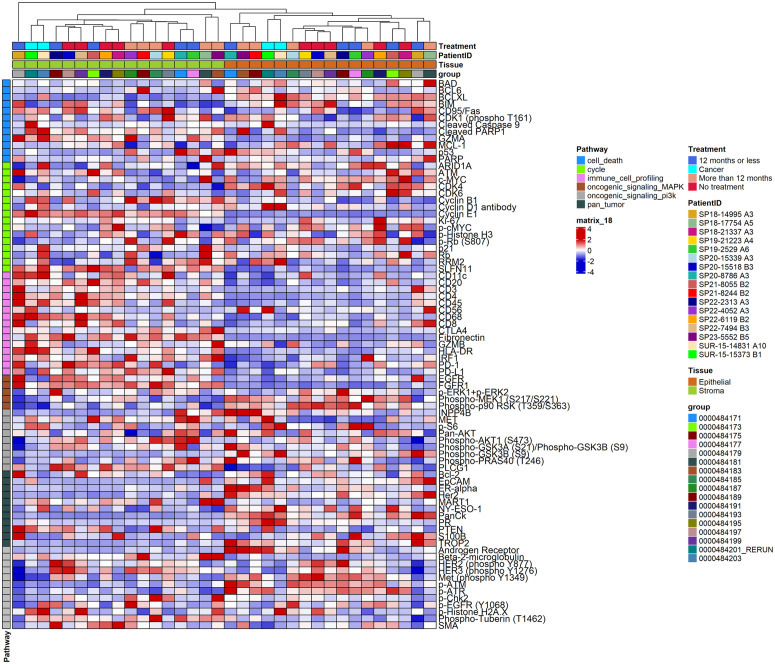
Unsupervised hierarchical clustering for benign samples only (nontreatment, STT, and LTT groups). STT: Short-term dihydrotestosterone treatment. LTT: Long-term dihydrotestosterone treatment.

To spatially investigate protein expression levels among benign groups, we next examined samples using NanoString’s GeoMx DSP. ER expression was elevated in the STT and LTT groups compared to the nontreatment group (log2FC = 2.3, p = 0.03, and log2FC = 2.4, p = 0.03, respectively), with no difference observed between the STT and LTT groups. AR levels were higher in the LTT group than in the nontreatment group (log2FC = 1.3, p = 0.03); however, no significant differences were observed between the nontreatment and STT groups, nor between the STT and LTT groups. Ki-67 expression was decreased in the LTT group relative to the nontreatment group (log2FC = −0.9, p = 0.02). A similar trend was observed between the STT and nontreatment groups, with a marginal difference (log2FC = 0.8, p = 0.06). Additionally, no difference was observed between the STT and LTT groups (Fig 4).

Among the cell cycle-related markers, p53 levels were markedly elevated in both the STT group (log2FC = 2.1, p = 0.01) and the LTT group (log2FC = 2.3, p = 0.01) compared to the nontreatment group, with no significant difference observed between short- and long-term DHT therapies. p21 levels were slightly decreased in the STT group but significantly elevated in the LTT group (log2FC = 0.9, p = 0.02) relative to baseline levels in the nontreatment group. A significant difference was also observed between the STT and LTT groups (log2FC = 1.0, p = 0.01). Cyclin-dependent kinase 6 (CDK6) levels showed a slight increase in the STT group compared to the nontreatment group but significantly decreased in the LTT group (log2FC = −0.5, p = 0.02). Expression of the proto-oncogene Inositol polyphosphate 4 phosphatase (INPP4B) was higher in the LTT group (log2FC = 1.0, p = 0.03), though this increase was not statistically significant in the STT group. Phosphorylated cMYC (p-cMYC) levels were inversely associated with the duration of treatment. Statistically significant differences were observed between the nontreatment group and the STT (log2FC = −0.4, p = 0.04) and LTT groups (log2FC = −0.6, p = 0.02). Phosphorylated Chk2 (p-Chk2), a DNA damage responder, was lower in the STT group (log2FC = −0.5, p = 0.04) but higher in the LTT group. While there was no difference between the nontreatment and LTT groups, a statistically significant difference was noted between the STT and LTT groups (log2FC = 1.0, p = 0.04). Finally, cCas9 levels were higher in the LTT group compared to the nontreatment group (log2FC = 0.7, p = 0.01).

The differences in the expression of immunologic markers were predominantly observed between the nontreatment and LTT groups. CD45 expression was lower in the LTT group (log2FC = −0.9, p = 0.01) but similar in the STT group. B-cell lymphoma 6 (BCL6) expression was higher in the LTT group compared to the nontreatment group (log2FC = 1.4, p = 0.01) but showed no change in the STT group. CD68 levels were lower in the LTT group (log2FC = −0.6, p = 0.006) compared to the nontreatment group. Although a gradual decrease in CD68 expression was observed in the STT group, the difference was not significant when compared to the nontreatment group. Other inflammatory markers, including CD3, CD20, and CD11c, showed no significant differences in expression between the groups (Table 1).

**Table 1:** Summary of Biomarker Expression in Benign Samples. The STT and LTT groups exhibit significant changes in biomarker expression compared to the nontreatment group, measured on a two-fold logarithmic scale. STT: Short-term dihydrotestosterone treatment. LTT: Long-term dihydrotestosterone treatment.

Marker	STT	LTT
ER	log2FC = 2.3, p = 0.03	log2FC = 2.4, p = 0.03
AR	n/a	log2FC = 1.3, p = 0.03
Ki67	n/a	log2FC = −0.9, p = 0.02
P53	log2FC = 2.1, p = 0.013	log2FC = 2.3, p = 0.0006
P21	n/a	log2FC = 0.9, p = 0.021
CDK6	n/a	log2FC = −0.5, p = 0.02
INPP4B	n/a	log2FC = 1.0, p = 0.026
p-cMYC	log2FC = −0.4, p = 0.04	log2FC = −0.6, p = 0.02
p-Chk2	log2FC = − 0.5, p = 0.04	n/a
CD45	n/a	log2FC = −0.9, p = 0.01
BCL6	n/a	log2FC = 1.4, p = 0.002
CD68	n/a	log2FC = − 0.6 p = 0.006
cCas9	n/a	log2FC = 0.7, p = 0.0062

### Cancer samples (Intra-tumoral comparison)

**Fig 5:**
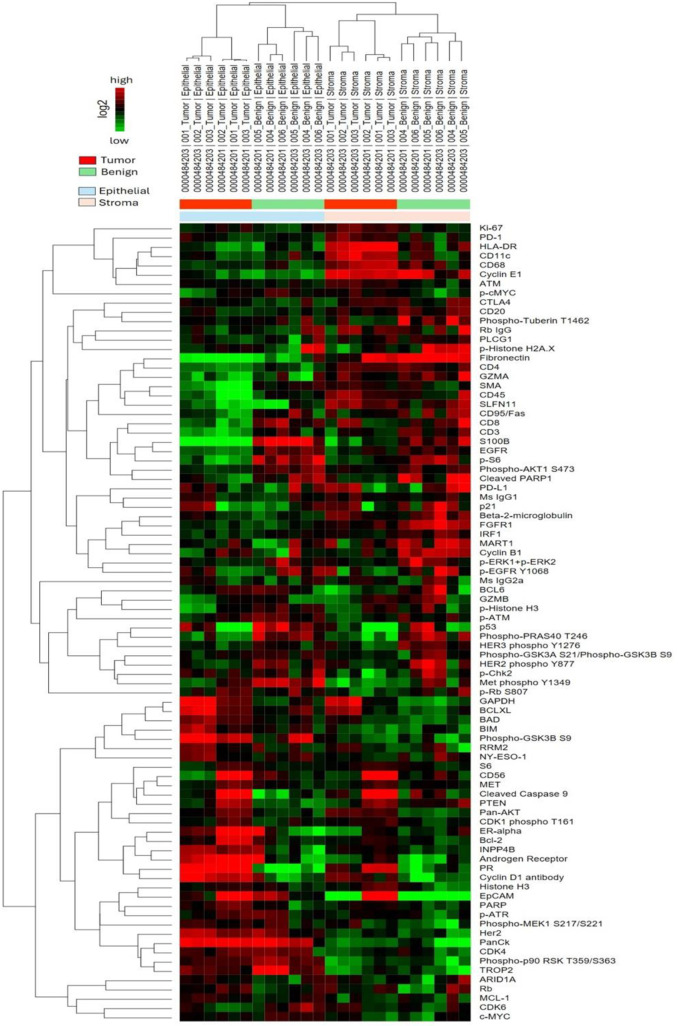
Unsupervised hierarchical clustering for BC samples only. Each 3 ROIs from a tumor or surrounding benign epithelium were collected within two BC samples. BC: Breast Carcinoma. ROIs: Region of Interest.

The analysis of three ROIs, each from the tumor and surrounding benign breast epithelium, revealed a significant decrease in S100 calcium-binding protein B (S100B) (log2FC = −4.6, p = 0.01), smooth muscle actin (SMA) (log2FC = −2.1, p = 0.001), and p-S6 (log2FC = −2.2, p = 0.03) levels in the carcinoma compared to the benign epithelial component. Conversely, AR (log2FC = 1.7, p = 0.02), PR (log2FC = 6.4, p = 0.03), and cyclin D1 (log2FC = 2.4, p = 0.009) levels were increased. Phospho-AKT1 and pan-AKT levels were marginally decreased in the tumor samples (log2FC = −0.9, p = 0.05). Immunologic biomarker levels, such as CD45 (log2FC = −1.2, p = 0.01) and CD3 (log2FC = −1.5, p = 0.02), were also decreased in the neoplastic epithelium. Similar to the benign LTT group, INPP4B expression was increased in the tumor samples (log2FC = 1.1, p = 0.01) (Fig 5). The tumor stroma showed elevated levels of pan-AKT (log2FC = 1.1, p < 0.001), cyclin D1 (log2FC = 1.1, p = 0.015), CD11c (log2FC = 2.1, p = 0.004), HLA-DR (log2FC = 1.7, p = 0.005), ATM (log2FC = 1.1, p = 0.015), CD68 (log2FC = 1.7, p = 0.02), and INPP4B (log2FC = 0.2, p = 0.03) compared to the surrounding benign stroma.

## Discussion

Among our patient cohort, nearly 80% underwent GAS, with nipple-sparing surgery being the most commonly chosen procedure. Most patients opt for chest reconstruction rather than total mastectomy. Young patients (<18 years), who constitute a small percentage (10%), exhibit no differences in morphological parameters or immunostaining results. Incidental precancerous lesions are rare, occurring in less than 1% of our samples. Most patients (85%) have received DHT treatment before surgery, including those with incidental carcinoma. Patients with incidental carcinoma did not undergo prior breast imaging, despite one having a strong family history of BC. Currently, no standard guidelines exist for incorporating imaging studies before GAS. Patients with a family history of breast carcinoma or genetic predisposition should receive, at a minimum, appropriate imaging and risk assessment. Among morphological features, aside from atrophy, secretory changes are the most notable findings associated with hormonal treatment. Key histological changes in the LTT group include apocrine snouting, luminal secretions, and cellular shedding.

Immunohistochemical and NanoString DSP analyses of our samples revealed partially overlapping results for ER and AR expression levels. Both AR and ER expression levels were elevated in the treatment groups compared to patients who had not received hormonal therapy before surgery. Immunohistochemistry showed that ER expression in large ductal epithelium elevated in the LTT group, with no differences observed among groups in peripheral breast tissue. In contrast, DSP analysis of lobular breast epithelium demonstrated higher ER levels in the LTT group compared to the nontreatment group. AR levels were increased in the LTT group compared to the nontreatment group, as confirmed by both methodologies. While immunohistochemistry detected a difference in AR levels between the STT and LTT groups, no difference was observed with NanoString DSP, indicating heterogeneity in AR expression across the samples. In a previous study, ER and AR exhibited the highest positivity rate and intensity in the lobular epithelium of FTM transgender patients with 24–47 months of testosterone exposure and subsequently decreased after >48 months of exposure [9]. Neither our cohort nor the previous study included a longitudinal component, i.e., a comparison of pre-DHT versus post-DHT treatment in the same individuals, due to practical limitations. Overall, the data suggest increased ER and AR expression with DHT treatment. However, inconsistencies between the nontreatment and the STT groups and between the STT and LTT groups may be attributed to the small sample size, undocumented testosterone or steroid use by patients before surgery, or technical and measurement limitations.

Furthermore, ER and AR co-expression demonstrated a strong correlation in the lactiferous ducts and peripheral breast tissue of the STT and LTT groups, assessed by immunohistochemistry. The role of ER and AR co-expression in the development of BC remains controversial [10]. A meta-analysis reports that ER-alpha and AR co-expression occurs in 75% of BC [11]. AR appears to be dichotomous depending on the BC subtype and ER-alpha status, the ER-alpha and AR pathways may antagonize or promote tumorigenesis. AR is expressed variably in histologically distinct subsets of mammary epithelial cells, particularly in apocrine carcinomas and apocrine metaplasia [12,13]. In ER-alpha-negative apocrine carcinoma, AR can stimulate cell growth [14], whereas in luminal ER-alpha-positive BC, AR has an antiproliferative role. Multiple *in vitro* studies on ER-positive BC cell lines have shown an antiproliferative effect of AR antagonism [15,16]. In the LTT group of benign samples, we observed the highest AR but the lowest Ki67 levels by NanoString DSP.

High DHT concentrations increase the expression of the *TP53* and *p21* genes in epithelial prostate cell lines [17]. Estrogens have also been shown to increase p53 protein levels in BC and lactating breast tissue through indirect mechanisms [18], including increased transcription and activation of the *c-myc* gene [19] and forming a triple complex of ER-alpha, p53, and MDM2 to protect p53 degradation in a ligand-dependent manner [20]. A common feature of these studies is that estrogen activation of ER-alpha leads to increased expression and cytoplasmic accumulation of p53 [21]. However, mutant p53 protein turnover is insensitive to estrogens. In our samples, p53 levels increased in benign samples treated with DHT and were not significantly different in the ER- and AR-positive tumor epithelium than surrounding normal tissue. In BC, p53 expression is more frequent (59%) when ER is absent than when ER is present (19%). Furthermore, p53 expression is associated with better survival in patients whose tumor did not express ER but worse survival in patients whose tumor expressed ER [22]. One of the limitations of our study is the absence of AR-positive and ER-negative cancer samples for comparative expression analysis.

Among the markers elevated in the LTT group of benign samples and cancer samples, INPP4B, known for its dual role in cancer biology, functions as both an oncogene and tumor suppressor. It acts as a negative regulator of the phosphoinositide 3-kinase (PI3K)/AKT signaling pathway, promoting cell proliferation, survival, migration, and metabolism [23]. In our samples, no significant differences in phospho-AKT levels were observed among groups. Pan-AKT showed a marginal decrease in STT and LTT samples, with a low fold change (log2FC < 0.5). In the tumor samples, Pan-AKT and phospho-AKT were also marginally decreased in the epithelial component, although Pan-AKT was significantly increased in the tumor stroma. In contrast to their known hyperactivity in BC development and progression, p-cMYC and CDK6 levels decreased in the LTT group of benign samples but not in cancer samples. Previous studies have shown that c-MYC levels are inhibited in ER-alpha-positive BC cell lines and cell lines treated with DHT [24], consistent with our findings in benign samples from the LTT group. CDK6, which binds to Cyclin D during the G1-to-S phase transition of the cell cycle, plays an essential role in the initiation, growth, and survival of many cancer types [25,26]. Cyclin D1 levels did not differ in benign samples but were elevated in both epithelium and stroma of cancer samples. The PI3K/AKT signaling pathway is likely involved in promoting Cyclin D1 upregulation in cancer samples [27].

Several immune pathway-related markers were either increased or decreased in benign and cancer samples. CD45 and CD68 levels were decreased in the LTT group of benign samples. Similarly, CD45, CD3, and CD8 levels were also lower in tumor epithelium than those in the benign epithelium from cancer samples. CD45 plays a key role in initiating T-cell receptor signaling by regulating the activation of protein-tyrosine kinases [28]. ER-positive BC is well-known for having less tumor-infiltrating lymphocytes (TIL) [29]. Interestingly, fewer TILs are associated with better survival in ER-positive BC, which is opposite to what is observed in ER-negative tumors [30]. The abundance of CD68 is also an adverse factor in ER-positive BC [31]. Our benign samples from LTT had lower CD68 levels, but DHT-treated BC samples had higher CD68 levels in tumor stroma.

In contrast to CD45 and CD68, BCL6 levels were elevated in the LTT group of benign samples. BCL6, a proto-oncogene, is required for the development of follicular T helper cells, which support germinal center formation [32] where plasma cells and memory B cells develop. BCL6 functions as a transcriptional repressor and may play an important role in BC progression [33] and resistance to CDK6 inhibitors [34]. Neither of the two ER- and AR-positive cancer samples exhibited elevated BCL6 levels in our analysis. Finally, the markers involved in the epithelial-mesenchymal transition, including S100B, SMA, and p-S6, were decreased in tumor epithelial samples compared to matching benign epithelium from the same sections. They can play distinct roles in the molecular subtypes of BC. For example, S100B has a favorable prognostic effect by suppressing the migratory capacity of ER-negative BC [35]; however, it acts as an adverse prognostic marker for ER-positive BC [36]. Similarly, low SMA levels detected via immunohistochemistry are associated with brain metastasis in triple-negative BC [37].

In conclusion, DHT treatment leads to significant morphological, hormonal, and molecular changes in both normal breast epithelium and breast cancer samples. Aside from atrophy, secretory changes, including apocrine snouting and cell shedding into the ductal lumens, are strongly associated with DHT treatment. Furthermore, DHT treatment appears to increase ER and AR expression in breast tissue with a strong expression correlation in both lactiferous ducts and lobular epithelium. Cell cycle-related and immunologic markers, including INPP4B and CD45, exhibit altered expression levels in the LTT group. Notably, the same markers display similar expression trends in ER- and AR-positive BC samples. These findings highlight the need for further research to clarify the role of these biomarkers in breast cancer development and prognosis.

## Supporting information

S1 File Combined Morphological and IHC Data: Combined Morphological and Immunohistochemistry Data. (XLSX)

S2 File IHC Scores computer image analysis: Immunohistochemistry Scores and computer image analysis. (XLSX)

S3 File DSP: Digital Spatial Profiling. (XLSX)
